# Impact of donor-to-recipient weight ratio on the hospital outcomes of pediatric heart transplantation

**DOI:** 10.1186/s43044-022-00276-8

**Published:** 2022-05-13

**Authors:** Mohammad Mahdavi, Tahmineh Tahouri, Avisa Tabib, Hooman Bakhshandeh, Ali Sadeghpour-Tabaei, Hossein Shahzadi, Nader Harooni

**Affiliations:** 1 Rajaie Cardiovascular Medical and Research Center, Iran University of Medical Science, Tehran, Iran; 2 Shahid Modarres Educational Hospital, Department of Pediatric Cardiology, Shahid Beheshti University of Medical Science, Tehran, Iran; 3 Heart Valve Disease Research Center, Rajaie Cardiovascular Medical and Research Center, Iran University of Medical Science, Tehran, Iran

**Keywords:** Cardiac transplantation, Donor selection, Transplant recipient

## Abstract

**Background:**

Identifying the factors that can influence the prognosis and final outcomes of pediatric heart transplantation is important and makes it possible to prevent complications and improve outcomes. Coordination of donor characteristics with the recipient in terms of sex, weight, body mass index (BMI), and body surface area (BSA) is an important factor that can influence the outcome of the transplantation. There is still no consensus regarding the role of discrepancy in anthropometrics between donors and recipients. The aim of this study was to investigate the relationship between donor and recipient weight mismatch on the early outcomes of pediatric heart transplantation. In this historical cohort study, 80 children who had underwent heart transplantation for the first time between 2014 and 2019 in Shahid Rajaie Cardiovascular Medical and Research Center in Tehran, Iran, were enrolled and divided into three groups according to donor-to-recipient weight ratio (0.8 < D/RW ≤ 1.5, 1.5 < D/RW ≤ 2.5, and 2.5 < D/RW). The early outcomes of transplantation, during the first post-transplant month, including right heart failure, renal failure, graft rejection, inotrope dependency, duration of intubation, length of ICU stay, death and requiring extracorporeal membrane oxygenation, were recorded through reviewing patient records.

**Results:**

Median donor-to-recipient BSA ratio was directly associated with higher vasoactive–inotropic score (*P* = 0.038), while no significant association was found between donor-to-recipient weight ratio and vasoactive–inotropic score (*P* = 0.07). No significant relationship was found between other outcomes and donor-to-recipient weight ratio or donor-to-recipient BSA ratio.

**Conclusions:**

Patients who require heart transplantation may also benefit from mismatch donors, especially in those with significant cardiomegaly.

## Background

In recent years, the number of annually performed pediatric heart transplants has been increasing [1]. However, the rate of transplantation is not as high as it needs to be [2]. According to US national data, from 2010 to 2015, there was a 31% increase in the heart transplant waiting list, while the annual increase in this surgery was only 28% [3]. Of all performed transplantations, the heart transplantation waiting list has the highest mortality. This gap between the number of performed transplantations and the number of required transplantations will result in efforts to accept graft tissues which are not ideal in terms of quality and properties by current criteria [4, 5].

Heart transplantation is the latest treatment in patients with severe heart failure who remain symptomatic despite standard medical therapy and are estimated to survive less than 1 year [6]. Pediatric heart transplantation accounts for 14% of all heart transplantations and improves the quality of life of these children and their families; however, it also has complications [7, 8]. The most important complications of heart transplantation are graft rejection, pulmonary hypertension, and infection. Despite improvements in early survival, through advancement of immunosuppressive therapy as well as surgical techniques and pre- and postoperative care, the lack of donated tissue is a major problem in these patients, which leads to a long waiting list and high mortality rate during this period [1]. In recent years, due to the lack of donated tissue, many studies have been conducted to increase the number of tissues and to accept marginal tissues as acceptable. These efforts include the use of older donated hearts or hearts with longer ischemic time [9]. In these studies, the use of Inotropes for the donor, type of mechanism that led to the donor’s death, duration of ischemia in the donor, and need for cardiopulmonary resuscitation in the donor are among the factors influencing the donor tissue to be considered marginal [10]. Coordination of donor characteristics with the recipient in terms of sex, weight, body mass index (BMI), and body surface area (BSI) is an important factor that can influence the outcome of the transplantation [11].

Identifying the factors that can influence the prognosis and final outcomes of pediatric heart transplantation is important and makes it possible to prevent complications and improve outcomes [12, 13]. There is still no consensus regarding the role of discrepancy in anthropometrics between donors and recipients. Accordingly, the aim of this study was to investigate the relationship between donor and recipient weight mismatch on the early outcomes of pediatric heart transplantation in a referral center in cardiovascular diseases in Iran. If it is found that the size mismatch between donors and recipients does not affect the early outcomes of heart transplantation, larger and even adult hearts can be used for this purpose.

## Methods

### Design

This observational study was conducted as a historical cohort. Census method was used to calculate the sample size, and 80 patients under 16 years old who had undergone a first-time heart transplantation between 2014 and 2019 in Shahid Rajaie Cardiovascular Medical and Research Center (RCMRC), a tertiary care hospital for cardiovascular patients in Tehran, Iran, were enrolled. The study protocol was approved in the review board and ethics committee of RCMRC. Patients' files were reviewed, and age, gender, type of heart disease leading to transplantation, familial history, weight, and body surface area (BSA) were recorded. Donor-to-recipient weight and BSA ratio was also calculated. Patients were divided based on donor-to-recipient weight ratio (D/RW) into three groups (0.8 < D/RW ≤ 1.5, 1.5 < D/RW ≤ 2.5, and 2.5 < D/RW). Donated hearts with a donor-to-recipient weight ratio of less than 0.8 were not used for transplantation in any of patients due to reports of poorer outcomes. Pre-transplant severe infection or sepsis, end organ damage, long-term intubation, and requiring extracorporeal membrane oxygenation (ECMO) were also considered as the exclusion criteria.

Early outcomes of transplantation during the first post-transplant month including the presence and severity of right heart failure, renal failure, graft rejection, inotrope dependency, duration of intubation (based on hours), length of intensive care unit (ICU) stay (by day), requiring ECMO and death were recorded after reviewing the patients' records.

### Definition of outcomes

Renal failure was considered as urine volume less than 0.5 cc/kg/hour or glomerular filtration rate less than normal range based on age. Graft rejection was detected based on echocardiographic data (new mitral regurgitation or decreased left ventricular function) and/or laboratory data (increased troponin). In heart transplantation, some degree of right ventricular dysfunction is expected because of preexisting elevated pulmonary vascular resistance of the recipient. This right ventricular dysfunction is usually less than moderate and does not last for more than several days.

In the current study, moderate-to-severe right ventricular dysfunction was defined based on echocardiographic findings (severe tricuspid regurgitation, tricuspid annular plane systolic excursion (TAPSE) > two standard deviations lower than expected for age and right ventricular Sa < 7 cm/s) at daily intervals until the 7th day after transplantation. Inotropic dependency was also defined based on vasoactive–inotropic score [VIS = dopamine dose (µg/kg/min) + dobutamine dose (µg/kg/min) + milrinone dose (µg/kg/min) × 10 + epinephrine dose (µg/kg/min) × 100 + norepinephrine dose (µg/kg/min) × 100 + vasopressin dose (U/kg/min) × 10000]. Whereas most heart transplant receivers need some inotropic support in early post-op, VIS > 13 is defined as inotrope dependency.

### Statistical Analysis

Normality of the interval variables was assessed using one-sample Kolmogorov–Smirnov test. Data were presented as median (inter-quartile range) for skewed interval and frequency (percentage) for categorical variables. Categorical variables were compared between D/R mismatch groups using chi-square for trend test. Skewed quantitative variables were compared between the study groups via Kruskal–Wallis test. For the statistical analysis, IBM SPSS Statistics 22 for Windows (IBM Inc., Armonk, NY) was used. *P* values less than 0.05 were considered statistically significant.

## Results

### Background characteristics of patients

Of these patients, 46 (57.5%) were male and 34 (42.5%) were female. The median age of patients at the time of transplantation was 133.50 months (inter-quartile range [IQR] 84–156 months). The median weight of the patients was 29.50 kg (IQR 17.25–38.75 kg). The median BSA ratio was 1.07 m^2^ (IQR 0.76–1.26 m^2^). Cause of heart failure was dilated cardiomyopathy in 66 patients (83%), restrictive cardiomyopathy in 3 patients (3.8%), congenital heart disease in 7 patients (9%), and other disease in 4 patients (5%). One patient had concomitant dilated cardiomyopathy and congenital heart disease.

The median time of intubation was 8 [6–15] hours. The median length of the ICU stay was also 7 (6 to 11.57) days. Familial history of cardiomyopathies of any types was present in 17 patients (21.3%). Median donor-to-recipient weight ratio was 1.75 and median donor-to-recipient BSA ratio was 1.42 (Table 1).

**Table 1:** Baseline characteristics of patients and variables studied on admission according to donor-to-recipient weight mismatch

Variable	Total	Groups based on D/R weight			*P* value
		0.8 < D/RW ≤ 1.5	1.5 < D/RW ≤ 2.5	D/RW > 2.5	
		(*n* = 30)	(*n* = 34)	(*n* = 16)	
Age (month)	133.5 (84–156)	155 (108.25–168.75)	132 (67.5–155.25)	99 (78.75–122.25)	0.009
*Gender*					
Male	46 (75.5%)	17 (56.7%)	22 (64.7%)	7 (43.8%)	0.553
Female	34 (42.5%)	13 (43.3%)	12 (35.3%)	9 (56.3%)	
Family history	17 (21.3%)	4 (13.3%)	11 (32.4%)	2 (12.5%)	0.720
Weight (kg)	19.5 (16.25–22.75)	38.5 (19–58.25)	32.5 (17.75–38)	19.5 (16.25–22.75)	0.001
BSA (m^2)^	0.84 (0.69–0.9)	1.27 (0.81–1.64)	1.09 (0.79–1.23)	0.84 (0.69–0.9)	0.001
*Heart failure type*					0.582
DCM	66 (83%)	26 (87%)	27 (79%)	13 (81%)	
RCM	3 (4%)	1 (3%)	1 (3%)	1 (6%)	
Congenital	7 (9%)	2 (7%)	4 (12%)	1 (6%)	
Others	4 (5%)	1 (3%)	2 (6%)	1 (6%)	

Data presented as median (Q1–Q3) for interval and count (%) for categorical variables. The first quartile (Q1) is equal to the 25th percentile and the third quartile (Q3) is equal to the 75th percentile of the data

*D/R W*, donor-to-recipient weight ratio

### Patient outcomes

Right ventricular dysfunction was mild in 39 patients (48.8%), moderate in 27 patients (33.8%), and severe in 14 patients (17.5%). Twenty-six patients (32.5%) had renal failure. Thirty patients (37.5%) were inotrope dependent with vasoactive–inotropic score (VIS) of more than 13. In addition, 23 patients (28.8%) experienced graft rejection. Five patients (6.3%) died during the follow-up period, and 11 patients (13.8%) required ECMO.

### Relationship between patient outcomes and D/R weight and D/R BSA mismatch

There was a moderate relationship between age and D/R weight, so that the higher the D/R W, the younger the patient (*P* = 0.009). This means that at a younger age, the weight disproportion between the donor and the recipient is greater. Similar association was revealed between donor-to-recipient weight ratio and BSA (*P* < 0.001). Thus, the younger the age, the higher the D/R BSA (*P* < 0.001).

There was also a significant relationship between the weight and BSA of the transplant recipients and the D/R W (*P* = 0.001 for both), which means that the lower the patient's weight or BSA, the greater the mismatch between the D/R weight and D/R BSA. This suggests that at a lower age, the number of suitable donors for heart transplantation is limited. In terms of other characteristics, no significant relationship was found between gender and the two ratios (*P* = 0.64).

There was a significant relationship between inotrope dependency (vasoactive–inotropic score of more than 13) and D/R BSA (*P* = 0.038), while no significant association was found between D/R weight and VIS of more than 13 (*P* = 0.05).

In assessment of other outcomes including death, length of ICU stay, duration of intubation, severe right ventricular dysfunction, renal failure, graft rejection, requiring ECMO or death, no significant relationship was found between any of these outcomes and the two anthropometric ratios. As shown in Table 2, after dividing patients by donor-to-recipient mismatch into three groups including D/R weight between 0.8 and 1.5, between 1.5 and 2.5, and above 2.5, none of the transplant outcomes mentioned above was significantly different between groups (Table 2).

**Table 2:** Patient outcome based on weight mismatch groups

Outcome	Groups based on D/R mismatch			*P* value
	0.8 < D/RW ≤ 1.5	1.5 < D/RW ≤ 2.5	D/RW > 2.5	
	(*n* = 30)	(*n* = 34)	(*n* = 16)	
Renal failure	8 (26.7%)	11 (32.4%)	7 (43.8%)	0.25
Graft rejection	9 (30.0%)	11 (32.4%)	3 (18.8%)	0.51
Inotrope dependency*	8 (26.7%)	13 (38.2%)	9 (56.3%)	0.05
Death	2 (6.7%)	2 (5.9%)	1 (6.3%)	0.93
ECMO** need	3 (10.0%)	6 (17.6%)	2 (12.5%)	0.69
RV*** dysfunction				0.08
Mild	18 (60.0%)	17 (50.0%)	4 (25.0%)	
Moderate	8 (26.7%)	10 (29.4%)	9 (56.3%)	
Severe	4 (13.3%)	9 (56.3%)	3 (18.8%)	
Intubation time (hours)	8 (6–10)	8 (6–14)	10 (8–21.5)	0.26
ICU stay (days)	7 (6–10.25)	7 (6–12)	7.5 (7–14.25)	0.60

*Inotrope dependency was defined as vasoactive–inotropic score > 13

**Extracorporeal membrane oxygenation

***Right ventricle

## Discussion

In this study, we investigated the impact of anthropometrics donor–recipient mismatch on short-term outcomes of transplant recipients. Evaluated outcomes were death, graft rejection, right ventricular failure, renal failure, need for ECMO, inotrope dependency, duration of intubation, and length of ICU stay during the first month after transplantation.

The results of this study showed that the weight mismatch between donor and recipient did not affect the mentioned outcomes in patients. As in the univariate correlation analysis, none of the studied variables were associated with the donor-to-recipient weight mismatch as well as with the donor-to-recipient BSA mismatch. In other words, after dividing the patients based on the donor-to-recipient weight into three groups (between 0.8 and 1.5, between 1.5 and 2.5, and above 2.5), none of the outcomes were significantly different between these three groups. These results are in line with other studies that have been conducted on this field. As shown by Jayarajan et al., the use of low donor-to-recipient weight ratio did not influence median survival, but in female donor-to-male recipient group, lower donor-to-recipient weight ratio was associated with decreased median survival [14]. Tang et al. also found that in patients with low donor-to-recipient weight ratio, acute rejection during the first month after transplantation is less common; however, infants with donor-to-recipient weight ratio between 0.5 and 0.59 had lower 1-month survival.

There was no difference in survival of patients with donor-to-recipient weight ratio 0.6–0.79 and donor-to-recipient weight ratio 0.8–2.0, and thus they suggested that donor-to-recipient weight ratio between 0.6 and 0.8 has no adverse outcome on the survival of pediatric heart transplantation [15]. A similar result to the current study was observed in adult patients. In a study by Patel et al. performed on 15,284 patients receiving heart transplants, patients were divided into three groups based on donor-to-recipient weight ratio, less than 0.8, between 0.8 and 1.2, and above 1.2. In this study, among patients with a donor-to-recipient weight ratio of less than 0.8, 5-year survival was lower in recipients with high pulmonary vascular resistance, and similar results were observed in other patients [10].

On the other hand, in the current study if the donor-to-recipient weight ratio was less than 0.8, the donated tissue was not used for heart transplantation. This policy is due to the fact that in previous studies, the donor-to-recipient weight ratio of less than 0.8 is one of the risk factors for transplant rejection and donor heart failure. Tamisier et al. indicated that donor-to-recipient weight ratio less than 1 is a risk factor for donor heart failure and early mortality. In this study, from 1987 to 1994, 73 patients who underwent heart transplantation were retrospectively evaluated. Pulmonary hypertension before the transplantation, major inotropic support of the donor, and the donor-to-recipient weight ratio were risk factors for donor heart failure. Donor heart failure was seen in 50% of patients whom donor-to-recipient weight ratio was less than 1, 33% of patients with donor-to-recipient weight ratio between 1 and 1.6, and 7% of patients with donor-to-recipient weight ratio more than 1.6. They also concluded that patients, particularly with pulmonary hypertension, may benefit from the use of grafts with greater donor-to-recipient weight ratio [16].

In another study by Conway et al., a lower donor-to-recipient ratio was a risk factor for transplant rejection [3], whereas some studies have shown that undersized allografts with weight mismatch between donor and recipient more than 20% did not relate to increased mortality [17]. Sethi et al. showed that early and late mortality of patients who had received smaller allograft with donor and recipient weight difference of 30% to 46% were not different from other patients with donor and recipient weight difference less than 30% [18]; thus, data on the use of small allografts are conflicting.

Whereas matching donated tissue with the recipient is a complex process, race, age, and size of the donor are important determinants in this area. Our study showed that in patients who are candidates for heart transplantation, allografts which are not matched with the recipient in terms of weight can be used, especially allografts which are larger in size. Since most cases of brain death occur in adults who have a larger heart in size, this size mismatch has limited the number of heart transplantations. The results of this study will reduce this limitation; however, this study examined only early outcomes, and more studies are needed to evaluate the late consequences of using a larger allograft.

## Conclusions

According to this study, higher donor-to-recipient weight ratios had no adverse effect on early outcomes. Therefore, donor pools can be expanded using non-baby donors with weights not commensurate with weight of the recipient baby; thereby, mortality of many children who are waiting to receive a heart transplant will be reduced.

## Data Availability

The authors confirm that the data supporting the findings of this study are available within the article and its supplementary materials**.**

## Abbreviations

BSA: Body surface area
D/R W: Donor-to-recipient weight ratio
ECMO: Extracorporeal membrane oxygenation
ICU: Intensive care unit
TAPSE: Tricuspid annular plane systolic excursion
VIS: Vasoactive–inotropic score
